# Sociodemographic factors, clinical characteristics, outcomes and short-term follow-up in COVID-19 patients with new onset hyperglycemia and pre-existing diabetes on admission in a tertiary-care hospital in Bangladesh

**DOI:** 10.1371/journal.pone.0311508

**Published:** 2024-12-19

**Authors:** Nadira Sultana Kakoly, S. M. Kamrul Hasan, Mohammad Mahfuzul Hoque, Rifat Hossain Ratul, Md. Abdullah Saeed Khan, Dipak Kumar Mitra, Baki Billah

**Affiliations:** 1 North South University, Dhaka, Bangladesh; 2 Monash University, Clayton, Victoria, Australia; 3 Dhaka Medical College Hospital, Dhaka, Bangladesh; 4 National Institute of Preventive and Social Medicine, Dhaka, Bangladesh; Uttara Adhunik Medical College, BANGLADESH

## Abstract

**Background:**

COVID-19 has been linked to hyperglycemia and diabetes, with noteworthy variation in outcomes. This study aimed to compare the sociodemographic factors, clinical characteristics, and in-hospital and short-term post-discharge outcomes between COVID-19 patients with new onset hyperglycemia and pre-existing diabetes patients in tertiary care hospitals in Bangladesh.

**Methods:**

A prospective observational study was conducted among adult COVID-19 patients with new onset hyperglycemia or pre-existing diabetes admitted to the COVID-19 unit of Dhaka Medical College Hospital between April 2021 and October 2021. Patients were conveniently selected from indoors. Bivariate analysis was used to compare sociodemographic and clinical characteristics at admission and short-term outcomes. The Cox proportional hazard model was used to examine factors associated with time to death in the hospital. All statistical analyses were performed using Stata Version 17.

**Results:**

A total of 169 patients were included. Of these, 29 died in the hospital, and four left against medical advice. Out of the 136 survivors, 135 came for follow-up two weeks after discharge. At baseline, 30.18% of patients had new onset hyperglycemia, and 69.8% had pre-existing diabetes. The average age of patients was 56.38 ± 14.21 years, and 60.36% were male. A significantly higher proportion of COVID-19 patients with new onset hyperglycemia were smokers than those with pre-existing diabetes (p = 0.003). However, pre-existing diabetes was associated with higher lung involvement (p = 0.047) and comorbidities (p = 0.002). Age, income over 35,000 BDT (USD 335.5$), and a BMI over 25 kg/m^2^ emerged as significant predictors of prolonged hospital stay and mortality. Post-discharge follow-up indicated that new-onset hyperglycemia resolved in 8.89% of patients, whereas 19.26% continued to exhibit hyperglycemia, with smoking being a significant determinant of its persistence (p = 0.001).

**Conclusion:**

In conclusion, our investigation illuminates the clinical trajectory of new-onset hyperglycemia in the context of COVID-19 and reinforces the necessity for diligent monitoring and management post-discharge. Therefore, close monitoring and follow-up of COVID-19 patients is recommended for the early detection and management of hyperglycemia and the prevention of diabetes development in the long run.

## Introduction

The COVID-19 pandemic has affected over half a billion people and claimed over six million lives globally as of December 2022 [1]. In Bangladesh, nearly two million people contracted COVID-19 during this period, with a death toll of approximately 17 per 100,000 [2]. Although global vaccination efforts have reduced the spread and severity of COVID-19 [3], the long-term health impacts among survivors continue to persist. This includes physical and mental problems, as well as decreased quality of life in all dimensions, and immeasurable socioeconomic impacts [4–6]. Survivors of COVID-19 are also at a higher risk of developing chronic diseases such as diabetes, heart disease, neurological conditions, and psychological illness due to the involvement of multiple organ systems during the infection [7]. Long-time persistence of certain symptoms and development of new chronic diseases like diabetes mellitus has been documented in two nationwide studies conducted in Bangladesh [8, 9]. Diabetes Mellitus (DM) is one of the leading causes of non-communicable disease-related deaths worldwide [10]. People with DM are more vulnerable to infections and risk developing severe COVID-19 infections with increased fatality [11]. This is because COVID-19 can impair glucose metabolism, cause hyperglycemia, and weaken the immune system. The virus uses the envelope spike protein to bind to angiotensin-converting enzyme 2 (ACE2) receptors, which are abundant in metabolically active tissues of the body, including the pancreatic islet cells [12, 13]. This can impair islet function and lead to hyperglycemia and new-onset diabetes among COVID-19 patients [14]. COVID-19 survivors with DM are significantly more likely to have problems in mobility and may suffer moderate to severe pain than those without DM [15].

Studies have shown that the prevalence of new-onset hyperglycemia is significant among hospitalised COVID-19 patients [16, 17]. Pre-existing DM cases, new-onset DM and hyperglycemia are associated with increased mortality [14]. Both type 1 and 2 DM have been newly detected in COVID-19-affected individuals [18]. The duration of hospital stay was reported to be significantly lower among COVID-19 patients with new onset hyperglycemia compared to those with pre-existing DM in Wuhan Union Hospital, China [19]. However, few studies have prospectively followed COVID-19 patients with new hyperglycemia or DM to assess their outcomes and glycaemic status in low-resource settings like Bangladesh. Therefore, this study aimed to compare the sociodemographic factors and clinical characteristics of COVID-19 patients with new onset hyperglycemia and pre-existing diabetes mellitus (DM), to investigate the determinants of hospital stay duration among these patients, with a specific focus on identifying whether hyperglycemic status influences the length of hospitalization and outcomes and to conduct a short-term follow-up after discharge to assess glycaemic status in a large tertiary care hospital in Bangladesh.

## Methodology

### Study setting, participants, and duration

This prospective observational study was conducted in the COVID-19 unit of Dhaka Medical College Hospital in Bangladesh between April 2021 and October 2021. During this period, the baseline information was collected at admission to the hospital, and outcomes were assessed at discharge. A follow-up interview and assessment were done 14 days after the discharge again in the hospital. The study included adult (≥18 years) COVID-19 patients with either new-onset hyperglycemia or new-onset diabetes at their admission. COVID-19 was confirmed by RT-PCR, or rapid antigen tests, and chest high-resolution computed tomography (HRCT) scans. Patients who were critically ill, had severe mental disorders, type 1 diabetes, diabetes insipidus, Cushing’s syndrome, Acromegaly, or were unwilling to give consent were excluded from the study.

### Sample size calculation and follow-up

Taking proportions of death among patients with new-onset hyperglycemia (4.7%) and pre-existing diabetes (11.2%) with COVID-19 from Li et al. [19], and setting confidence intervals at 95% and power at 80%, the sample size was calculated using the following formula [20],

$$\text{n}=\frac{{\left[Z_{\alpha }\sqrt{p_{0}\left(1-P_{0}\right)}+Z_{\beta }\sqrt{P(1-P)}\right]}^{20}}{{\left(P_{0}-P\right)}^{2}}$$

**Fig 1:**
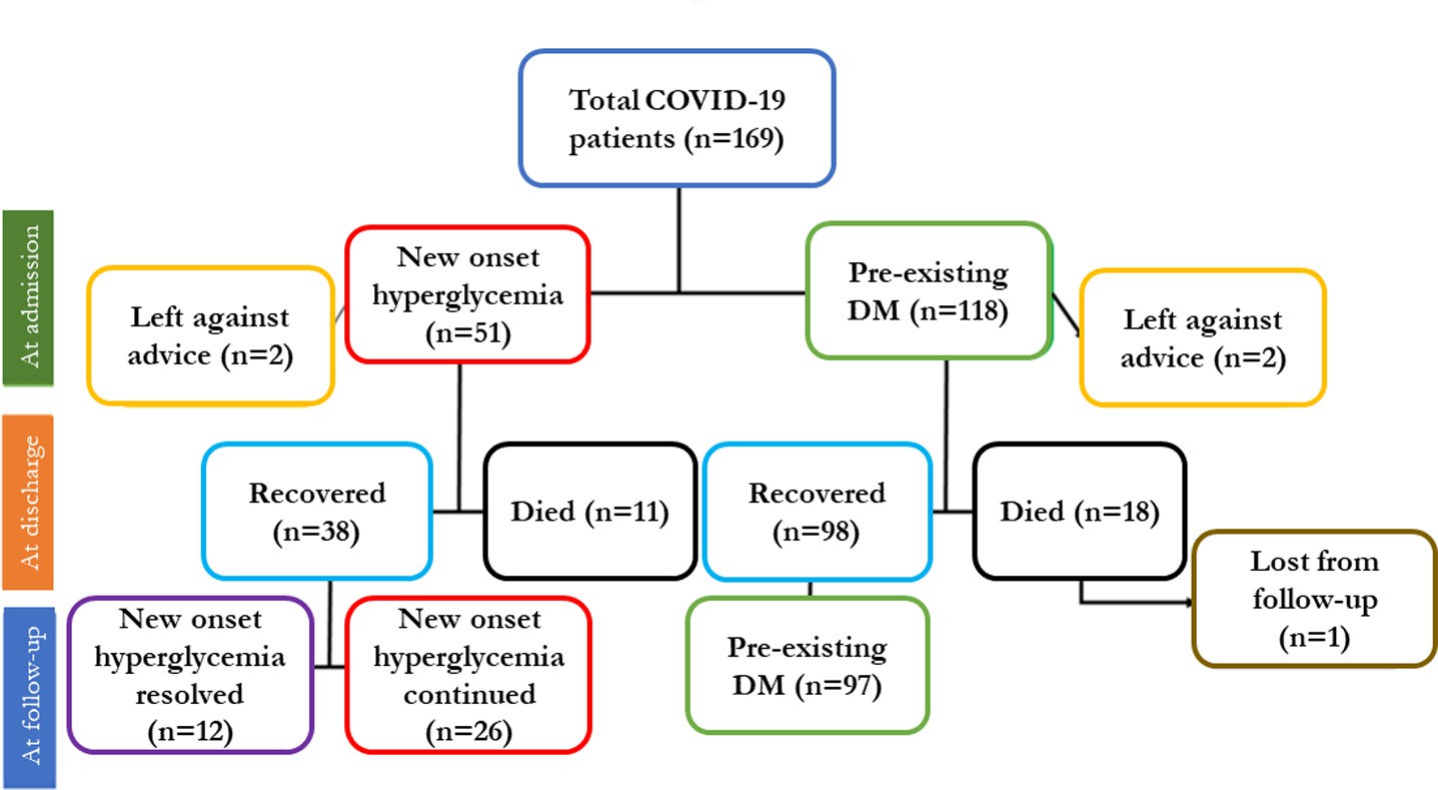
Flow chart showing patient selection, outcome at discharge and follow-up two weeks after discharge.

Here, p_0_ = proportion of death among pre-existing diabetes patients = 11.2%; p = proportion of death among new hyperglycemia patients = 4.7%; Z_α_ = standard normal deviate at 95% Confidence Interval = 1.96; Z_β_ = Standard normal deviate at 80% power = 0.84; Therefore, the calculated sample size, n = 109.35~ 109. Taking 10% non-response the final sample size was estimated to be 109+11 = 120 for each group. However, a total of 118 COVID-19 patients with pre-existing diabetes and 59 patients with new onset hyperglycemia could be included at admission (baseline) until mid-October 2021. Patients were approached consecutively from the wards. Those willing to participate were screened for exclusion criteria. Afterward, based on their history and baseline glycemic profile, new-onset hyperglycemia and pre-existing diabetes were defined and included. Those included were followed up until either discharge from the hospital or death in the hospital. Four patients left the hospital against medical advice, and 29 patients died in the hospital. The remaining 136 patients were advised to come for a follow-up visit 14 days after discharge, and 135 patients (over 99%) attended the follow-up visit (**Fig 1**).

### Data collection

Patients selected for data collection using a convenient sampling technique. A structured questionnaire was used for data collection. The questionnaire was formed after reviewing existing studies on similar topics [16, 17, 19, 21–24] and contextualized after considering existing resources at hand. The socioeconomic and demographic profile, COVID-19 vaccination history, severity of COVID-19 on admission, past history of diabetes and other comorbidities (hypertension, chronic obstructive pulmonary disease, bronchial asthma, chronic heart disease, and cancer), and treatments given during hospitalization were collected from the admission records and treatment charts. The following glycaemic measurements were collected at baseline: HbA1c, fasting blood sugar (FBS), blood sugar 2 hours after breakfast (2ABF), and random blood sugar (RBS). The FBS and 2ABF were repeated during the follow-up visit. The authors had access to information about patients during data collection. However, data collection forms were anonymized so that individual patients could not be identified after the data collection process.

### Definitions

The following diagnostic criteria were used to define new onset hyperglycemia and pre-existing diabetes based on the admission glycaemic measurements and history of diabetes and following guidelines from the American Diabetic Association Standards of Care in Diabetes-2023 Abridged for Primary Care Providers [25].

#### New onset hyperglycemia

We defined *new onset hyperglycemia* as the absence of a previous diabetes diagnosis, indicated by an HbA1c level of <6.5%, and the presence of fasting blood sugar (FBS) levels ≥7.0 mmol/l, 2 hours after meal blood sugar (2ABF) levels ≥11.1 mmol/l, or random blood sugar (RBS) levels ≥11.1 mmol/l, accompanied by classic symptoms of hyperglycemia or a hyperglycemic crisis.

#### Previously undiagnosed DM

We considered patients with previously undocumented diabetes who had an HbA1c ≥6.5% plus either one or more of FBS ≥7.0 mmol/l or 2ABF ≥11.1 mmol/l or RBS ≥11.1 mmol/l as having undiagnosed diabetes. The glycemic features according to **ADA guidelines** which deem patients as having diabetes if they have either HbA1c ≥6.5% or FBS ≥7.0 mmol/l, 2ABF ≥11.1 mmol/l, and RBS ≥11.1 mmol/l.

#### New onset hyperglycemia resolved

Patients diagnosed with hyperglycemia at admission who were subsequently found to have FBS <7.0 mmol/l and 2ABF <11.1 mmol/l at follow-up (14 days after discharge).

#### Pre-existing DM

These included both previously undiagnosed DM and previously diagnosed DM.

#### Previously diagnosed DM

previously diagnosed and documented as having DM by a physician or were taking anti-diabetic medication.

### Statistical analysis

Descriptive statistics were expressed as frequency (percentage) for categorical variables and mean ± standard deviation (SD) or median (interquartile range [IQR]) for continuous variables. Bivariate analysis was conducted using the chi-square test and Fisher’s exact test for categorical variables, and the independent samples t-test and one-way analysis of variance (one-way ANOVA) were used for normally distributed continuous variables. For non-normally distributed data, the respective analysis was done using the Mann-Whitney U test and Kruskal-Wallis test. A multivariable Cox proportional hazards model was used to determine the factors associated with duration of hospital stay with in-hospital mortality of COVID patients with diabetes. Patients enrolled at admission were entered into the analysis and they were censored upon death in the hospital, or discharge with advice or against medical advice. The factors included in the model were based on clinical judgement, the outcome of bivariate analysis, and those that were found to have an influence on successive loadings in the model. Collinear variables were excluded. A p-value of <0.05 was considered significant for all analyses, and all statistical analysis was conducted using Stata Version 17 (StataCorp LLC, Texas, USA).

### Ethical consideration

This study was approved by the Institutional Review Board/Ethical Review Committee of North South University (#2020/OR-NSU/IRB/1116). All the procedures were conducted in accordance with the guidelines laid out by the Declaration of Helsinki. Informed written consent was taken from participants before inclusion.

## Results

Out of 169 COVID-19 patients enrolled in the study, 51 (30.18%) had new onset hyperglycemia and 118 (68.82%) had pre-existing diabetes at admission. The average age of the patients was 56.38 years, with 60.36% being male and 78.57% being married. The median monthly family income was 25,000 BDT (240$), and 51.83% of the patients came from rural areas. Socio-demographic profiles were similar between patients with new onset hyperglycemia and pre-existing DM (**Table 1**).

**Table 1:** Sociodemographic characteristics of patients stratified by new onset hyperglycemia and pre-existing diabetes at admission.

Variable	New onset hyperglycemia	Pre-existing DM	Total	p-value
**n (%)**	51 (30.18)	118 (69.82)	169 (100.00)	
***Socio-demographic profile***				
**Age (years)**	54.82 ±16.28	57.06 ±13.24	56.38 ±14.21	0.349
**Sex**				
**Male**	36 (70.59)	66 (55.93)	102 (60.36)	0.074
**Female**	15 (29.41)	52 (44.07)	67 (39.64)	
**Marital Status**				
**Married**	42 (82.35)	90 (76.92)	132 (78.57)	0.430
**Single (Unmarried/Separated/Divorced)**	9 (17.65)	27 (23.08)	36 (21.43)	
**Education (years)**	10 (5–13)	10 (5–12)	10 (5–12)	0.146
**Residence**				
**Urban**	21 (41.18)	58 (51.33)	79 (48.17)	0.228
**Rural**	30 (58.82)	55 (48.67)	85 (51.83)	
**Monthly income (BDT) [US dollars]**	25000 (20000–40000)	30000 (15000–40000)	25000 (15000–40000)	0.487
	[240 (192–383.5)]	[288 (144–383.5)]	[240 (144–383.5)]	
**Monthly income categories in BDT (US Dollars)**				
**≤35000 (≤335.5 USD)**	36 (70.59)	86 (72.88)	122 (72.19)	0.760
**>35000 (>335.5 USD)**	15 (29.41)	32 (27.12)	47 (27.81)	

Data was presented as mean ±SD, median (IQR) and n (%) where appropriate. The proportions presented shows column percentage. p-value was determined by Chi-square test, Fisher’s exact test, independent samples *t* test and Mann-Whitney U test where appropriate. Significant p-values are shown in bold.

BDT: Bangladeshi Taka;

### Clinical characteristics and personal habits stratified by glycemic status at admission

**Table 2** provides a comprehensive analysis of clinical characteristics and personal habits among COVID-19 patients categorized by new onset hyperglycemia and pre-existing diabetes at admission. The cohort had a diverse body mass index (BMI) distribution, with 49.70% falling within the normal range (18.5–24.9 kg/m^2), 44.97% classified as overweight or obese (BMI ≥ 25 kg/m^2), and a small fraction (5.33%) underweight (BMI < 18.5 kg/m^2). A majority of patients (70.41%) were afflicted with severe COVID-19, reflecting the critical nature of the cases managed in the clinic. The median lung involvement, represented as a percentage, was higher in patients with pre-existing diabetes (48%) compared to those with new onset hyperglycemia (45%), indicating a statistically significant difference (p = 0.047).

**Table 2:** Clinical characteristics and personal habits of patients stratified by new-onset hyperglycemia and pre-existing diabetes at admission.

Variable	New onset Hyperglycemia	Pre-existing DM	Total	p-value
**n (%)**	51 (30.18)	118 (69.82)	169 (100.00)	
**Body Mass Index (kg/m** ^**2**^ **)**				
**<18.5**	3 (5.88)	6 (5.08)	9 (5.33)	0.786
**18.5–24.9**	27 (52.94)	57 (48.31)	84 (49.70)	
**≥ 25**	21 (41.18)	55 (46.61)	76 (44.97)	
***COVID-related information***				
**Severity of COVID**				
**Moderate**	13 (25.49)	37 (31.36)	50 (29.59)	0.443
**Severe**	38 (74.51)	81 (68.64)	119 (70.41)	
**Lung involved (%)**	45 (40–70)	48 (30–60)	46.5 (30–60)	**0.047**
**COVID-19 vaccine**				
**Taken**	6 (11.76)	15 (12.71)	21 (12.43)	0.864
**Not taken**	45 (88.24)	103 (87.29)	148 (87.57)	
***Personal habits***				
**Current smoker**				
**Yes**	17 (33.33)	16 (13.68)	33 (19.6)	**0.003**
**No**	34 (66.67)	101 (86.32)	135 (80.36)	
**Exercise habit**				
**Regular**	9 (18.37)	14 (12.28)	23 (14.11)	0.306
**Irregular/none**	40 (81.63)	100 (87.72)	140 (85.89)	
***Comorbidities***				
**Hypertension**				
**Present**	16 (31.37)	70 (60.87)	86 (51.81)	**<0.001**
**Absent**	35 (68.63)	45 (39.13)	80 (48.19)	
**Chronic heart disease**				
**Present**	3 (6.00)	15 (13.16)	18 (10.98)	0.277
**Absent**	47 (94.00)	99 (86.84)	146 (89.02)	
**COPD**				
**Present**	2 (3.92)	8 (7.08)	10 (6.10)	0.726
**Absent**	49 (96.08)	105 (92.92)	154 (93.90)	
**Bronchial Asthma**				
**Present**	8 (15.69)	24 (21.62)	32 (19.75)	0.378
**Absent**	43 (84.31)	87 (78.38)	130 (80.25)	
**Cancer**				
**Present**	1 (1.96)	1 (0.90)	2 (1.23)	0.532
**Absent**	50 (98.04)	110 (99.10)	160 (98.77)	
**Any other comorbidity**				
**Present**	24 (48.00)	81 (72.97)	105 (65.22)	**0.002**
**Absent**	26 (52.00)	30 (27.03)	56 (34.78)	
***Admission vitals***				
**Pulse (/min)**	83 ±8	85 ±13	85 ±12	0.268
**Systolic blood pressure (mmHg)**	119 ±15	121 ±13	121 ±14	0.292
**Diastolic blood pressure (mmHg)**	75 ±8	76 ±8	76 ±8	0.315
**SpO**_**2**_ **(%)**	94.49 ±3.41	93.97 ±4.70	94.12 ±4.35	0.474

Data was presented as mean ±SD, median (IQR) and n (%) where appropriate. The proportions presented shows column percentage.

p-value as determined by Chi-square test, Fisher’s exact test, and Mann-Whitney U test where appropriate. Significant p-values are shown in bold.

COPD: Chronic obstructive pulmonary disease; COVID: Coronavirus disease; DM: Diabetes mellitus;

Only 12.43% of the study population had received a COVID-19 vaccine, which could be reflective of the overall vaccination rates within the study’s time period. A substantial association was found between new onset hyperglycemia and current smoking (33.33%), contrasting with a lower prevalence (13.68%) in patients with pre-existing diabetes (p = 0.003).

Hypertension was the most prevalent comorbidity (51.81%), particularly pronounced in the pre-existing diabetes group (60.87% vs. 31.38% in new onset hyperglycemia, p<0.001). Patients received a range of treatments, including low molecular weight heparin (91.72%), remdesivir (52.07%), and dexamethasone (93.49%), indicative of the clinical protocols for severe COVID-19 cases during the study period.

**Table 3** delineates laboratory findings, administered treatments, and outcomes for COVID-19 patients stratified by glycemic status at admission.

**Table 3:** Laboratory findings, treatments and outcome of patients stratified by new-onset hyperglycemia and pre-existing diabetes at admission.

Variable	New onset hyperglycemia	Pre-existing DM	Total	p-value
**n (%)**	51 (30.18)	118 (69.82)	169 (100.00)	
***Investigations***				
**HbA1c (%)**	5.58 ±0.67	8.82 ±2.64	7.80 ±2.68	**<0.001**
**FBS (mmol/l)**	8.36 ±2.98	11.25 ±4.85	10.37 ±4.56	**<0.001**
**2ABF (mmol/l)**	11.54 ±4.40	13.64 ±4.79	13.00 ±4.76	**0.008**
**RBS (mmol/l)**	13.06 ±3.63	15.78 ±7.83	14.95 ±6.93	**0.020**
***Treatments given***				
**LMWH**				
**Yes**	47 (92.16)	108 (91.53)	155 (91.72)	1.000
**No**	4 (7.84)	10 (8.47)	14 (8.28)	
**Remdesivir**				
**Yes**	32 (62.75)	56 (47.46)	88 (52.07)	0.068
**No**	19 (37.25)	62 (52.54)	81 (47.93)	
**Dexamethasone**				
**Yes**	49 (96.08)	109 (92.37)	158 (93.49)	0.508
**No**	2 (3.92)	9 (7.63)	11 (6.51)	
**Duration of hospital stay (days)**	13 (8–20)	13 (10–18)	13 (9–18.5)	0.322
**Outcome**				
**Alive**	38 (77.55)	98 (84.48)	136 (82.42)	0.285
**Died**	11 (22.45)	18 (15.52)	29 (17.58)	

Data was presented as mean ±SD, median (IQR) and n (%) where appropriate. The proportions presented shows column percentage.

p-value as determined by Chi-square test, Fisher’s exact test, independent samples *t* test and Mann-Whitney U test where appropriate. Significant p-values are shown in bold.

FBS: Fasting blood sugar; 2ABF: Blood sugar 2 hour after breakfast; LMWH: Low molecular weight heparin.

#### Laboratory findings

Upon admission, patients exhibited an average SpO2 of 94.12%. The glycemic profile showed elevated HbA1c (7.80%), fasting blood sugar (FBS; 10.37 mmol/l), 2-hour post-breakfast blood sugar (2ABF; 13.00 mmol/l), and random blood sugar (RBS; 14.95 mmol/l). Notably, these glycemic parameters were significantly higher in the new onset hyperglycemia group compared to those with pre-existing diabetes, highlighting the impact of acute illness on glycemic control (p<0.05).

#### Treatments administered

The majority of patients received low molecular weight heparin (LMWH; 91.72%), while more than half were treated with remdesivir (52.07%), and a significant proportion with dexamethasone (93.49%). The choice of treatments reflects the prevailing medical protocols for managing severe COVID-19 cases during the study period.

#### Hospital stay and mortality

The median duration of hospital stay was consistent across groups at 13 days. Mortality was observed in 17.58% of the cohort, with a slightly higher, albeit non-significant, incidence in the new onset hyperglycemia group (22.45%) compared to those with pre-existing diabetes (15.52%).

### Post-discharge glycemic profiles and related characteristics in COVID-19 patients

Table 4 synthesizes the follow-up assessments conducted two weeks post-discharge from the hospital for COVID-19 patients, examining the persistence of hyperglycemic states and associated clinical characteristics.

**Table 4:** Characteristics of patients stratified by status of diabetes at follow-up.

Variable	New-onset Hyperglycemia resolved	New-onset Hyperglycemia continued	Pre-existing diabetes	p-value
**n (%)**	12 (8.89)	26 (19.26)	97 (71.85)	
**Age (years)**	44.67 ±12.85	56.19 ±16.28	55.73 ±13.20*	**0.031**
**Body Mass Index (kg/m** ^**2**^ **)**				
**<18.5**	0 (0.00)	1 (3.85)	4 (4.12)	0.989
**18.5–24.9**	6 (50.00)	14 (53.85)	51 (52.58)	
**≥ 25**	6 (50.00)	11 (42.31)	42 (43.30)	
**Severity of COVID**				
**Moderate**	2 (16.67)	10 (38.46)	33 (34.02)	0.455
**Severe**	10 (83.33)	16 (61.54)	64 (65.98)	
**Lung involved (%)**	56 (40–80)	42.5 (32.5–62.5)	41 (30–50)	0.169
**Current smoker**				
**Yes**	1 (8.33)	11 (42.31)	10 (10.42)	**0.001**
**No**	11 (91.67)	15 (57.69)	86 (89.58)	
**Exercise habit**				
**Regular**	1 (8.33)	6 (24.00)	11 (11.83)	0.285
**Irregular/none**	11 (91.67)	19 (76.00)	82 (88.17)	
**Hypertension**				
**Present**	3 (25.00)	10 (38.46)	58 (61.05)	**0.015**
**Absent**	9 (75.00)	16 (61.54)	37 (38.95)	
**Chronic heart disease**				
**Present**	0 (0.00)	3 (11.54)	8 (8.51)	0.577
**Absent**	11 (100.00)	23 (88.46)	86 (91.49)	
**COPD**				
**Present**	0	1 (3.85)	2 (2.15)	0.646
**Absent**	12 (100.00)	25 (96.15)	91 (97.85)	
**Bronchial Asthma**				
**Present**	2 (16.67)	3 (11.54)	19 (20.88)	0.578
**Absent**	10 (83.33)	23 (88.46)	72 (79.12)	
**Cancer**				
**Present**	1 (8.33)	0	0	0.093
**Absent**	11 (91.67)	26 (100.00)	91 (100.00)	
**Any comorbidity**				
**Present**	5 (45.45)	14 (53.85)	64 (70.33)	0.102
**Absent**	6 (54.55)	12 (46.15)	27 (29.67)	
**Systolic blood pressure (mmHg) at follow-up**	119 ±9	123 ±9	122 ±15	0.707
**Diastolic blood pressure (mmHg) at follow-up**	80 ±6	78 ±5	78 ±7	0.651
**FBS at follow-up (mmol/l)**	6.25 ±0.98	7.06 ±1.85	10.51 ±3.82*^!^	**<0.001**
**2ABF at follow-up (mmol/l)**	7.44 ±0.86	9.62 ±1.84	10.75 ±3.29*	**0.008**
**Duration of hospital stay (days)**	13 (9.5–17.5)	13.5 (9–20)	14 (10–19)	0.827

Data was presented as mean ±SD, median (IQR) and n (%) were appropriate. The proportions presented shows column percentage.

p-value as determined by Chi-square test, Fisher’s exact test, one-way analysis of variance (ANOVA) and where appropriate. Post-hoc analysis was carried out using Bonferroni adjustments (p-value was significant at <0.05 level compared to *new-onset DM resolved, and ^!^new-onset DM continued). Significant p-values are shown in bold.

COPD: Chronic obstructive pulmonary disease; COVID: Coronavirus disease; DM: Diabetes mellitus; FBS: Fasting blood sugar; 2ABF: Blood sugar 2 hour after breakfast;

### Follow-up glycemic status

A bifurcation in glycemic control was noted among the cohort: 8.89% of patients with new onset hyperglycemia demonstrated normalized glucose levels, while 19.26% continued to experience hyperglycemia, and the remaining 71.85% maintained their pre-existing diabetic condition.

### Demographic and clinical insights

Patients who experienced resolution of hyperglycemia were significantly younger (mean age 44.67 years) compared to those with persistent hyperglycemia (mean age 56.19 years) and pre-existing diabetes (mean age 55.73 years). Smoking prevalence was markedly higher (42.31%) in patients with persistent hyperglycemia, suggesting a link between smoking and prolonged dysglycemia post-COVID-19 recovery. Hypertension was most common in patients with pre-existing diabetes (61.05%), indicating potential interplay between chronic conditions and acute infectious processes.

### Hospitalization and outcomes

The duration of hospital stay did not significantly differ across groups, pointing to a consistent treatment duration irrespective of glycemic status. Interestingly, the resolution of hyperglycemia did not correlate with a shorter hospital stay, indicating other factors may influence recovery time.

A comparison of the baseline characteristics of COVID-19 patients with diabetes who died and those who recovered and were discharged from the hospital is presented in **S1 Table**. Patients who died had higher age (p = 0.003), higher monthly family income (>35000 BDT; >333.5$) (p = 0.015), higher BMI (p = 0.025), lower SpO2 at admission (p = 0.035), higher proportion of chronic heart disease (p = 0.019) and COPD (p = 0.009), and lower duration of hospital stay (p = 0.021) than those who were alive.

### Multivariable cox regression analysis of hospital stay and mortality in COVID-19 patients with diabetes

Table 5 presents the results from a multivariable Cox regression analysis that identified significant predictors of in-hospital mortality among COVID-19 patients with diabetes.

**Table 5:** Cox regression analysis exploring factors associated time to in-hospital deaths of COVID-19 patients with DM.

Factors	Reference Category	Adjusted HR (95%CI)	p-value
**Age**		1.05 (1.01–1.09)	**0.006**
**Sex (Female)**	**Male**	1.02 (0.37–2.81)	0.963
**Monthly income category (>35000 BDT [>335.5 USD])**	**≤35000 BDT [>335.5 USD]**	2.57 (1.06–6.25)	**0.036**
**COVID severity (Severe)**	**Moderate**	2.02 (0.39–10.25)	0.395
**Smoking (Yes)**	**No**	1.82 (0.60–5.56)	0.292
**Exercise (Irregular/None)**	**Regular**	1.43 (0.36–5.70)	0.608
**New onset hyperglycemia**	**Pre-existing DM**	1.42 (0.56–3.59)	0.459
**Any other comorbidity (Present)**	**Absent**	0.46 (0.18–1.19)	0.110
**Body Mass Index (kg/m** ^**2**^ **)**			
** <18.5**	**18.5–24.9**	2.79 (0.73–10.65)	0.133
** ≥25**	**18–5–24.9**	2.90 (1.05–7.99)	**0.039**
**Remdesivir (Not given)**	**Given**	1.17 (0.49–2.83)	0.723
**LMWH (Given)**	**Not given**	2.33 (0.11–47.91)	0.585
**Dexamethasone (Not given)**	**Given**	4.42 (0.18–107.01)	0.361

Significant p-values are shown in bold.

BDT: Bangladeshi Taka; COPD: Chronic obstructive pulmonary disease; COVID: Coronavirus disease; DM: Diabetes mellitus; FBS: Fasting blood sugar; 2ABF: Blood sugar 2 hour after breakfast; LMWH: Low molecular weight heparin.

### Significant predictors of mortality

The analysis, adjusting for sex, severity of COVID-19, smoking status, exercise habits, diabetes type, comorbidities, and treatments (remdesivir, LMWH, dexamethasone), revealed that age, family income, and BMI were significant prognostic factors. Each additional year of age was associated with a 5% increase in the hazard of mortality (Adjusted Hazard Ratio [HR]: 1.05; 95% Confidence Interval [CI]: 1.01–1.09, p = 0.006). Higher family income (HR: 2.57; 95% CI: 1.06–6.25, p = 0.036) and a BMI above 25 kg/m^2 (HR: 2.90; 95% CI: 1.05–7.99, p = 0.039) were linked to an increased hazard of death, highlighting socioeconomic and obesity-related disparities in COVID-19 outcomes.

While other factors such as sex, smoking, and exercise were not statistically significant, their inclusion in the model is reflective of their potential influence on patient outcomes. Notably, the presence of comorbidities surprisingly suggested a protective trend, though not statistically significant (HR: 0.46; 95% CI: 0.18–1.19, p = 0.110).

## Discussion

Our study conducted in a resource-constrained tertiary clinic setting reveals a notable incidence of new onset hyperglycemia (30.18%) at admission among COVID-19 patients, with 19.26% persisting with hyperglycemia two weeks post-recovery. A significant correlation was established between smoking and the emergence of new onset hyperglycemia. Furthermore, a greater extent of lung involvement and hypertension was observed in patients with pre-existing diabetes. The risk of in-hospital death increased among older individuals, those overweight or obese, and those with a higher monthly income of 35000 BDT (335.5 USD).

The occurrence of new onset hyperglycemia in our cohort aligns with previous literature, such as the findings of Zhang et al. [17] and is higher than the prevalence reported by Shrestha et al. in a systematic review and meta-analysis [14]. This discrepancy could stem from our focused enrollment criteria, which prioritized patients with blood glucose abnormalities during this period.

The study found that patients with pre-existing diabetes had higher involvement of the lungs on HRCT scans and a higher proportion of hypertension. Our findings substantiate the association between long-standing diabetes and immune compromise, contributing to the observed heightened lung involvement, as discussed by Li et al. [19]. The concomitant hypertension in diabetic individuals may be attributable to the increased peripheral vascular resistance commonly seen in this demographic [26], a pattern echoed in our findings.

A noteworthy finding of our study is the link between smoking and new-onset hyperglycemia, suggesting that smoking may exacerbate the beta-cell dysfunction and inflammatory response induced by COVID-19, thereby prolonging hyperglycemia, as supported by Li et al. [19]. Our findings could be explained by nicotine’s known effects on body composition and insulin resistance [27]. Therefore, people with habits of smoking could have suffered the added inflammatory stress of COVID-19 further damaging pancreatic beta cells and causing hyperglycemia to persist.

In contrast to other reports [14, 17, 22, 28], our study did not observe a significant association between new onset hyperglycemia and mortality, possibly due to our comparison group being individuals with pre-existing diabetes rather than normoglycemic patients. This also applied to the lack of significant difference in disease severity, which is contrary to suggestions by other studies [22] of increased COVID-19 severity in hyperglycemic patients.

Our Cox Proportional Hazard model identifies age, income, and obesity as significant predictors of in-hospital mortality, resonating with the broader literature that cites these factors as independent risks for COVID-19 mortality [29–31].

The resolution of hyperglycemia in 8.89% of patients post-discharge, predominantly among younger and non-smoking individuals, contrasts with the persistence of hyperglycemia in others. While BMI did not emerge as a distinguishing factor in our study, contrasting with findings by Farag et al. [18], Montefusco et al. [28] suggest that hyperglycemia can persist for months post-COVID-19, potentially leading to overt diabetes. Our short-term follow-up limits the scope to predict diabetes development, highlighting the need for extended follow-up in future research.

Long COVID, characterized by sustained metabolic disruptions post-SARS-CoV-2 infection, remains a concern. Metabolic pathway abnormalities in survivors, as found by Li et al. [32], may continue beyond six months post-discharge. The potential diabetogenic effect of SARS-CoV-2, through its interaction with the ACE2 receptor expressed in key metabolic tissues [33], underscores the imperative for vigilant post-recovery monitoring of COVID-19 patients to enable early diabetes detection and management, thereby mitigating further complications.

### Limitation and strength

This study is one of the earliest to explore the metabolic implications such as new-onset hyperglycemia and pre-existing diabetes, on COVID-19, in hospitalised patients with a short-term follow-up at two weeks after discharge in a low-resource clinical setting. However, the study has some limitations, including a small sample size with data collected from a single centre and a short follow-up time. Moreover, we could not fully assess the impact of smoking–particularly the number of cigarettes smoked- with hyperglycaemia among these patients due to lack of information. A close monitoring of blood glucose dynamics longitudinally over a prolonged period was not possible. Hence, we could not definitely identify new onset diabetes. Exploration of serological markers in relation to new onset hyperglycemia was not possible either.

## Conclusion

The study highlights critical aspects of metabolic complications in COVID-19, demonstrating significant associations between new onset hyperglycemia, pre-existing diabetes, and patient outcomes. The persistence of hyperglycemia in nearly one-fifth of the patients post-discharge, especially among smokers, points to the need for targeted glycemic control interventions in this population. Given the significant predictors of mortality and prolonged hospitalization identified, there is a compelling case for the development of stratified care pathways. These should particularly focus on high-risk groups, such as older individuals, those with higher BMI, and patients from higher income brackets. However, the findings should be interpreted with caution, given the study’s limitations.

## Recommendations

Recommendations for future research include longitudinal studies to assess the long-term metabolic consequences of COVID-19 and assessment of the efficacy of integrated smoking cessation programs in improving glycemic control among survivors.

## Supporting information

S1 Table Characteristics of participants in relation to outcome at discharge. (DOCX)
