# Supplementary material for: Sociodemographic factors, clinical characteristics, outcomes and short-term follow-up in COVID-19 patients with new onset hyperglycemia and pre-existing diabetes on admission in a tertiary-care hospital in Bangladesh
Source: PLoS One. 2024 Dec 19;19(12):e0311508. doi: 10.1371/journal.pone.0311508 (PMC11658473; doi:10.1371/journal.pone.0311508)
Supplement: S1 Table — (DOCX) [file pone.0311508.s001.docx]

**S1 Table. Characteristics of participants in relation to outcome at discharge**

| **Variable** | **Recovered** | **Died** | **p-value** |
| --- | --- | --- | --- |
| **n (%)** | 136 (82.42) | 29 (17.58) |  |
| ***Socio-demographic profile*** |  |  |  |
| **Age (years)** | 54.98 ±14.13 | 63.55 ±13.24 | **0.003** |
| **Sex** |  |  |  |
| Male | 78 (57.35) | 21 (72.41) | 0.133 |
| Female | 58 (42.65) | 8 (27.59) |  |
| **Marital Status** |  |  |  |
| Married | 107 (79.26) | 22 (75.86) | 0.685 |
| Single | 28 (20.74) | 7 (24.14) |  |
| **Education (years)** | 10 (5 – 12) | 6 (5 – 12) | 0.294 |
| **Residence** |  |  |  |
| Urban | 65 (49.62) | 12 (41.38) | 0.422 |
| Rural | 66 (50.38) | 17 (58.62) |  |
| **Monthly income in thousands (BDT)** | 22.5 (15.5 – 30) | 30 (15 – 50) | 0.238 |
| **Monthly income category in BDT (US dollars)** |  |  |  |
| ≤35000 (≤335.5 USD) | 105 (77.21) | 16 (55.17) | **0.015** |
| >35000 (>335.5 USD) | 31 (22.79) | 13 (44.83) |  |
| ***Nutritional status*** |  |  |  |
| **Body Mass Index (kg/m^2^)** |  |  |  |
| <18.5 | 5 (3.68) | 4 (13.79) | **0.028** |
| 18.5 – 24.9 | 72 (52.94) | 9 (31.03) |  |
| ≥ 25 | 59 (43.38) | 16 (55.17) |  |
| ***COVID-related information*** |  |  |  |
| **Severity of COVID** |  |  |  |
| Moderate | 45 (33.09) | 3 (10.34) | **0.014** |
| Severe | 91 (66.91) | 26 (89.66) |  |
| **Lung involved (%)** | 42 (30 – 60) | 58 (45 – 70) | **0.025** |
| **COVID vaccine** |  |  |  |
| Taken | 18 (13.24) | 1 (3.45) | 0.201 |
| Not taken | 118 (86.76) | 28 (96.55) |  |
| ***Personal habits*** |  |  |  |
| **Current smoker** |  |  |  |
| Yes | 23 (17.04) | 9 (31.03) | 0.084 |
| No | 112 (82.96) | 20 (68.97) |  |
| **Exercise habit** |  |  |  |
| Regular | 18 (13.74) | 3 (10.71) | 0.668 |
| Irregular/none | 113 (86.26) | 25 (89.29) |  |
| ***Comorbidities*** |  |  |  |
| **Hyperglycemia / DM** |  |  |  |
| New-onset hyperglycemia | 38 (27.94) | 11 (37.93) | 0.285 |
| Pre-existing DM | 98 (72.06) | 18 (62.07) |  |
| **Hypertension** |  |  |  |
| Present | 71 (52.99) | 14 (50.00) | 0.774 |
| Absent | 63 (47.01) | 14 (50.00) |  |
| **Chronic heart disease** |  |  |  |
| Present | 11 (8.33) | 7 (25.00) | **0.019** |
| Absent | 121 (91.67) | 21 (75.00) |  |
| **COPD** |  |  |  |
| Present | 4 (3.03) | 5 (17.86) | **0.009** |
| Absent | 128 (96.97) | 23 (82.14) |  |
| **Bronchial Asthma** |  |  |  |
| Present | 25 (19.23) | 5 (17.86) | 1.000 |
| Absent | 105 (80.77) | 23 (82.14) |  |
| **Cancer** |  |  |  |
| Present | 1 (0.77) | 1 (3.57) | 0.324 |
| Absent | 129 (99.23) | 27 (96.43) |  |
| **Any comorbidity** |  |  |  |
| Present | 84 (65.12) | 18 (64.29) | 1.000 |
| Absent | 45 (34.88) | 10 (35.71) |  |
| ***Treatments given*** |  |  |  |
| **LMWH** |  |  |  |
| Yes | 123 (90.44) | 28 (96.55) | 0.468 |
| No | 13 (9.56) | 1 (3.45) |  |
| **Remdesivir** |  |  |  |
| Yes | 71 (52.21) | 17 (58.62) | 0.547 |
| No | 65 (47.79) | 12 (41.38) |  |
| **Dexamethasone** |  |  |  |
| Yes | 126 (92.65) | 28 (96.55) | 0.691 |
| No | 10 (7.35 | 1 (3.45) |  |
| ***Investigations*** |  |  |  |
| **SpO_2_ (%)** | 94.44 ±4.11 | 92.55 ±5.33 | **0.035** |
| **HbA1c (%)** | 7.87 ±2.64 | 7.67 ±2.92 | 0.721 |
| **FBS (mmol/l)** | 10.33 ±4.58 | 10.95 ±4.69 | 0.516 |
| **2ABF (mmol/l)** | 13.11 ±4.73 | 12.95 ±5.14 | 0.868 |
| **RBS (mmol/l)** | 14.82 ±7.22 | 15.85 ±5.57 | 0.482 |
| **Duration of hospital stay (days)** | 14 (10 – 20) | 13 (6 – 15) | **0.021** |

Table footnotes:

Data was presented as mean ±SD, median (IQR) and n (%) were appropriate. The proportions presented shows column percentage.

p-value as determined by Chi-square test, Fisher’s exact test, independent samples *t* test and Mann-Whitney U test where appropriate. Significant p-values are shown in bold.

BDT: Bangladeshi Taka; COPD: Chronic obstructive pulmonary disease; COVID: Coronavirus disease; DM: Diabetes mellitus; FBS: Fasting blood sugar; 2ABF: Blood sugar 2 hour after breakfast; LMWH: Low molecular weight heparin
